# Management of permanent teeth with necrotic pulps and open apices according to the stage of root development

**DOI:** 10.4317/jced.54287

**Published:** 2017-11-01

**Authors:** Hugo Plascencia, Mariana Díaz, Gerardo Gascón, Susana Garduño, Carlos Guerrero-Bobadilla, Salvador Márquez-De Alba, Geovani González-Barba

**Affiliations:** 1 DDS, Endodontic Postgraduate Program, CUCS, University of Guadalajara, Mexico; 2 DDS, MSc, Endodontic Postgraduate Program, CUCS, University of Guadalajara, Mexico; 3 DDS, MSc, PhD, Endodontic Postgraduate Program, CUCS, University of Guadalajara, Mexico; 4 DDS, Endodontic Postgraduate Program, CUAltos, University of Guadalajara, Mexico

## Abstract

**Background:**

This paper analyzed the distribution of treatments for permanent teeth with necrotic pulps and open apices according to the stage of root development.

**Material and Methods:**

Dental records from all root canal procedures performed in permanent teeth with necrotic pulps and open apices over a period of 14 years by residents of the Speciality of Endodontics, University of Guadalajara, Mexico, were analized.

**Results:**

Records from 206 treatments were mainly divided into the following 3 different stages according to criteria described by Cvek: stage IV (n = 79, 38.3%), stage V (n = 66, 32%) and stage III (n = 53, 25.7%). Few cases involved the initial stages of root development (stages I and II) (n = 8, 3.8%). Such teeth were submitted to four different treatments: MTA apical barrier (n = 69), Ca(OH)2 replacements (n = 34), gutta-percha (n = 67) and a plug of Ca(OH)2/gutta-percha (n = 36). The teeth with intermediate root development (Cvek stage III) showed a predilection for the MTA apical barrier and Ca(OH)2 replacement techniques (*P* ≤ 0.001). Furthermore, the stage of root development did not influence the apical extent of the root filling.

**Conclusions:**

The finding of permanent teeth with necrotic pulp and open apices is not exclusive to young patients with an open apex. Moreover, teeth with fragile, irregular and divergent apical morphologies, such as Cvek’s stages´ I and II, were not common and may be considered to be unusual findings. The diverse endodontic procedures were reliable regardless of the stage of root development.

** Key words:**Incomplete root formation, Open apex, Epidemiological studies, Root development.

## Introduction

The endodontic treatment of permanent teeth with open apices and necrotic pulp is considered one of the most complex challenges for clinicians. Usually, these teeth are associated with young people who develop early cavities, have a morphological anomaly or have suffered a dental trauma that prematurely halts root development (1). However, adults with completely formed roots can develop an open apex due to certain pathological (e.g., external inflammatory root resorption) (2,3) or iatrogenic (over-instrumentation) (4) factors, that modify mature apical lumen diameter. Therefore, such teeth show thin radicular walls with increased susceptibility to fracture, and the wide lumen of the apical foramen makes it difficult to maintain the filling material inside the root canal system and impossible for proper mechanical cleaning to avoid excessive root weakness (5-7). All of these characteristics compromise the long term health of the tooth.

**Figure 1:**
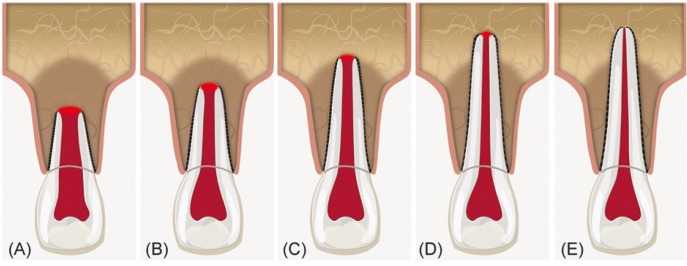
Schematic of Cvek’s stages of root development. (A) *Group I*, < 1/2 root length; (B) *Group II*, 1/2 root length; (C) *Group III*, 2/3 root length; (D) *Group IV*, wide open apical foramen and nearly completed root length; (E) *Group V*, closed apical foramen and completed root development. Groups I, II and III shows wide and divergent apical openings.

The assessment of dental maturity is a typical strategy employed by some dental specialities (e.g. forensic dentistry) (8,9), but few studies have examined the stage of root development in relation to the frequency of teeth with open apices and necrotic pulp (10-12). Several classification systems for the degree of root formation and maturation have been proposed (13-15), although Cvek’s (11) classification offers didactic radiographic characteristics with valuable clinical applications. Cvek’s classification describes the five stages of root development (Fig. 1): I = < 1/2 root length, II = 1/2 root length, III = 2/3 root length, IV = wide open apical foramen and nearly complete root length and, V = closed apical foramen and completed root development. While Cvek stage V describes mature, fully formed teeth, the remaining four stages describe teeth with open apices and a lack of apical constriction development but significant morphological differences. Cvek stages I, II and III indicate wide and divergent apical openings, the root canals are significantly wider in the buccal-lingual plane than in the mesio-distal plane, the terminal portion of the root is irregular and the apical foramen diameter is higher than the root canal lumen (16). In contrast, stage IV is associated with noticeable root length and convergent apical walls. Therefore, considering that endodontic procedure selection most likely depends on the maturity of the affected root, the goal of this study was to analyze the distribution of treatments in pemanent teeth with necrotic pulps and open apices according to the stage of root development in a Mexican sub-population.

## Material and Methods

-Case selection

The ethical committee of the Health Sciences University Center, University of Guadalajara, Mexico (C.I./13/2015), approved this epidemiological study. The study sample included patients who had received root canal procedures in permanent teeth with necrotic pulps and open apices performed by residents of the Speciality of Endodontics, University of Guadalajara, Mexico, from January 1, 2000, through December 31, 2013. The dental records eligible for this study included those for permanent teeth with pre-operational evidence of an open apex or cases where the master apical file corresponded to size #70- or larger. Clinical records with incomplete treatment sequence information, low quality/poor condition periapical radiographs, or descriptions of a revascularization/revitalization procedure were all excluded.

**Figure 2:**
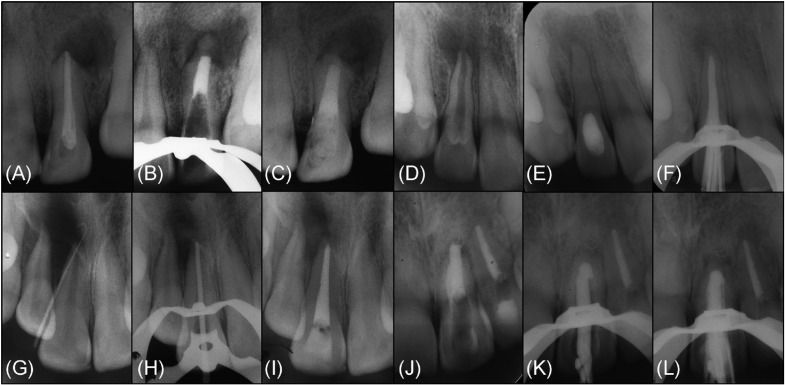
Radiographic sequence of the diverse endodontic techniques performed in permanent teeth with necrotic pulp and open apices included in this study. *Technique 1*: MTA apical barrier: (A) Pre-operative radiograph of tooth #22. (B) Radiographic verification of 0.5mm Ca(OH)2 powder placement as a resorbable extra-radicular barrier against which a 5 mm MTA was packed. (C) Final radiograph with a visible remaining canal filled with gutta-percha and the cervical third reinforced with composite resin. *Technique 2*: Ca(OH)2 replacements: (D) Pre-operative radiograph of tooth #12. (E) Radiographic aspect where the main canal seemed to have become calcified, which indicated that the entire canal had been adequately filled with Ca(OH)2. (F) After 2 Ca(OH)2 replacements over 9 months, a new hard tissue barrier was radiographically detected, and the root canal was subsequently filled with gutta-percha embedded in a calcium hydroxide sealer. *Technique 3*: Gutta-percha filling: (G) Pre-operative sinus tract radiograph of tooth #11. (H) Radiographic examination of size #80 master gutta-percha cone. (I) Final radiograph after 2-visit root canal treatment. *Technique 4*: Plug with Ca(OH)2/gutta-percha filling: (J) Pre-operative radiograph of tooth #21. (K) Radiographic examination of the apical adaptation of a master gutta-percha cone individualized through a softened filling technique. (L) Once the 0.5 mm Ca(OH)2 powder was placed as an apical plug, it was radiographically verified via the gutta-percha filling.

A total of 206 endodontics treatment performed in 192 patients were included in this study. For each dental record, a series of clinical and radiographic variables were compiled in a specialized database (SPSS 20.0, LEAD Technologies, Inc., Chicago, Illinois, USA). These variables included the following: age, sex, tooth location, tooth, stage of root development, etiologic agent, pulpal diagnosis, periapical diagnosis, radiographic periapical health, master apical file, intracanal dressing, number of Ca(OH)2 paste replacements, apical extent of root filling and obturation technique. After data collection, the 206 cases were grouped according to the endodontic technique modality performed (Fig. 2):

•Technique 1: MTA apical barrier (MTA)

•Technique 2: Ca(OH)2 replacements (CHR)

•Technique 3: Gutta-percha filling (GTP)

•Technique 4: Plug of Ca(OH)2/gutta-percha filling (CH-G)

-Radiographic analysis

To ensure data reliability, the review capabilities of two experienced endodontic specialists (H.P. and M.D.) were calibrated. For this purpose, without time constraints and under the same optimal observation conditions, each observer independently analyzed a total of 100 periapical radiographs of incisors, premolars and molars at diverse stages of root development and radiographic periapical health that were not part of this study. Cohen’s Kappa coefficient was used to determine the inter-observer concordance.

After the reviewers were calibrated, the diagnostic radiographs of all cases included in this study were delivered to each observer and analyzed under the aforementioned conditions. The Cvek classification criteria were used to determine the stage of root development (11) (Fig. 1). The periapical index scoring system (PAI) proposed by Orstavik *et al.* (17) was utilized to assess the radiographic periapical health. In multi-rooted teeth, the evaluation was limited to the root or roots with necrotic pulp and an open apex. Teeth treated due to trauma-induced dental displacement six months prior to the clinical presentation (e.g., subluxation, extrusion, lateral luxation or avulsion) or those involved in active orthodontic treatment were excluded from the periapical health radiographic analysis, because both situations can simulate the presence of a radiolucent periapical lesion. In case of disagreement between the two reviewers, a third reviewer made the final decision (C.G.-B.).

-Statistical analysis

Descriptive statistics were generated using SPSS 20.0 software for Windows (SPSS, LEAD Technologies, Inc., USA). The frequencies and percentages of the variables of interest (absolute and relative) were obtained and grouped in contingency tables according to the technique used. Subsequently, the distributions of such frequencies in each of the four procedures were compared using the χ2 test or Fisher’s exact test. The established significance level was *P* ≤ 0.05.

Next, the correlation between the stage of root development and the other variables were determined using a specific correlation coefficient according to each type of variable. For the eight nominal qualitative variables (sex, tooth location, tooth, etiologic agent, pulpal diagnosis, periapical diagnosis, intracanal dressing, and obturation technique), Cramer’s V was used. For the three ordinary qualitative variables (age, radiographic periapical health, and apical extent of root filling), Kendall’s Tau-B was employed. For the two qualitative variables (master apical file and number of Ca(OH)2 paste replacements), the Spearman coefficient was applied.

## Results

A total of 206 root canal procedures in permanent teeth with necrotic pulps and open apices were performed in 192 patients (female = 53.6%, male = 46.4%). Regarding the stage of root development, the inter-observer concordance obtained during the calibration process was k = 0.737, which indicate substantial concordance. The 206 cases were mainly distributed among 3 different Cvek stages (n = 198, 96.1%). Most cases were classified as Cvek stage IV (n = 79, 38.3%), followed by stage V (n = 66, 32%) and stage III (n = 53, 25.7%). Only a few cases involved the initial stages of root development (stages I and II), with 3 cases (1.5%) classified as stage I and 5 cases (2.4%) classified as stage II. In Table  Table 1,  Table 1 continue,  Table 1 continue-1,  Table 1 continue-2,  Table 1 continue-3, the general results for all variables are described in detail.

**Table 1:**
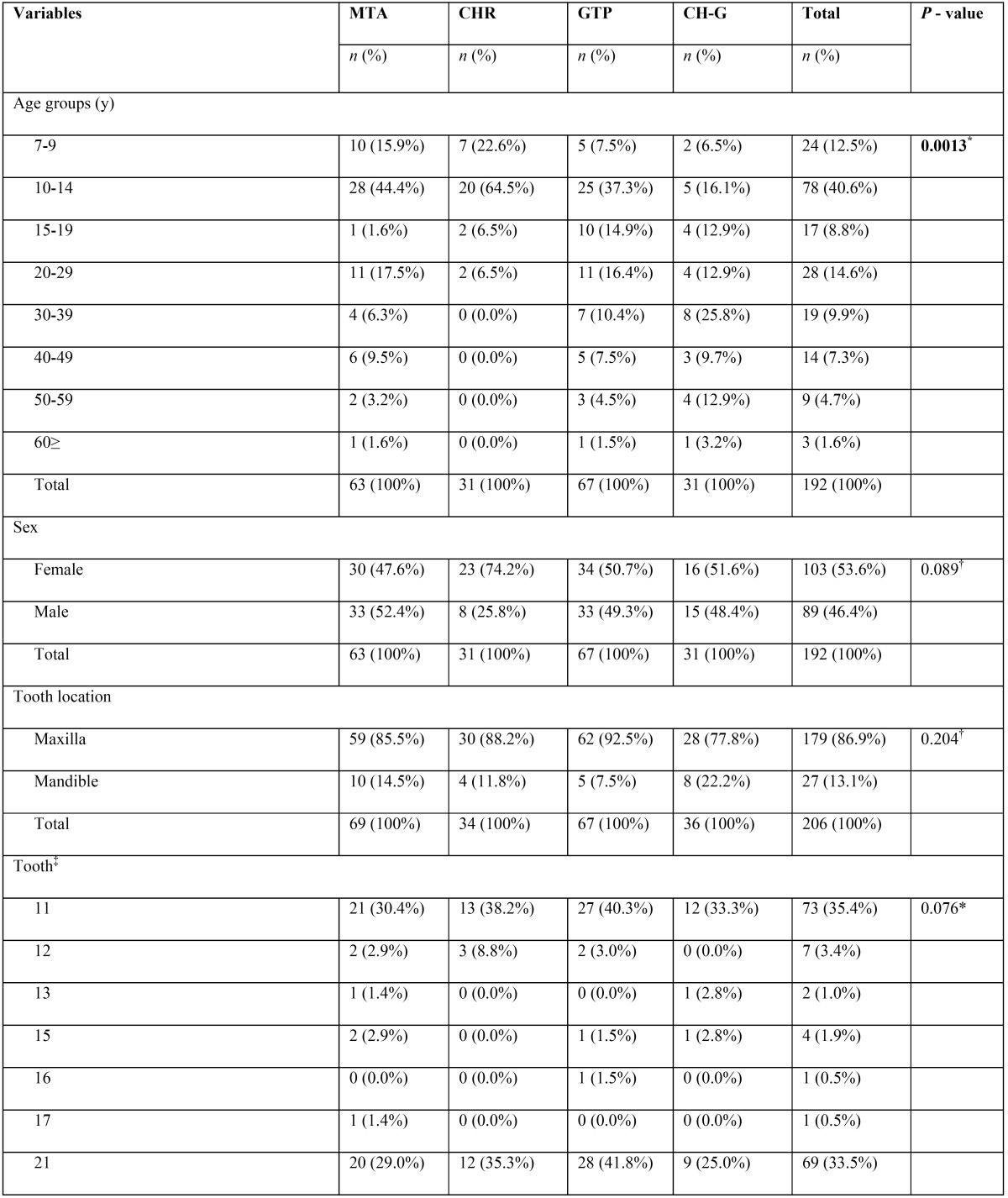
Distribution of the variables (n = 14) according to the endodontic technique modality applied.

**Table 1 continue:**
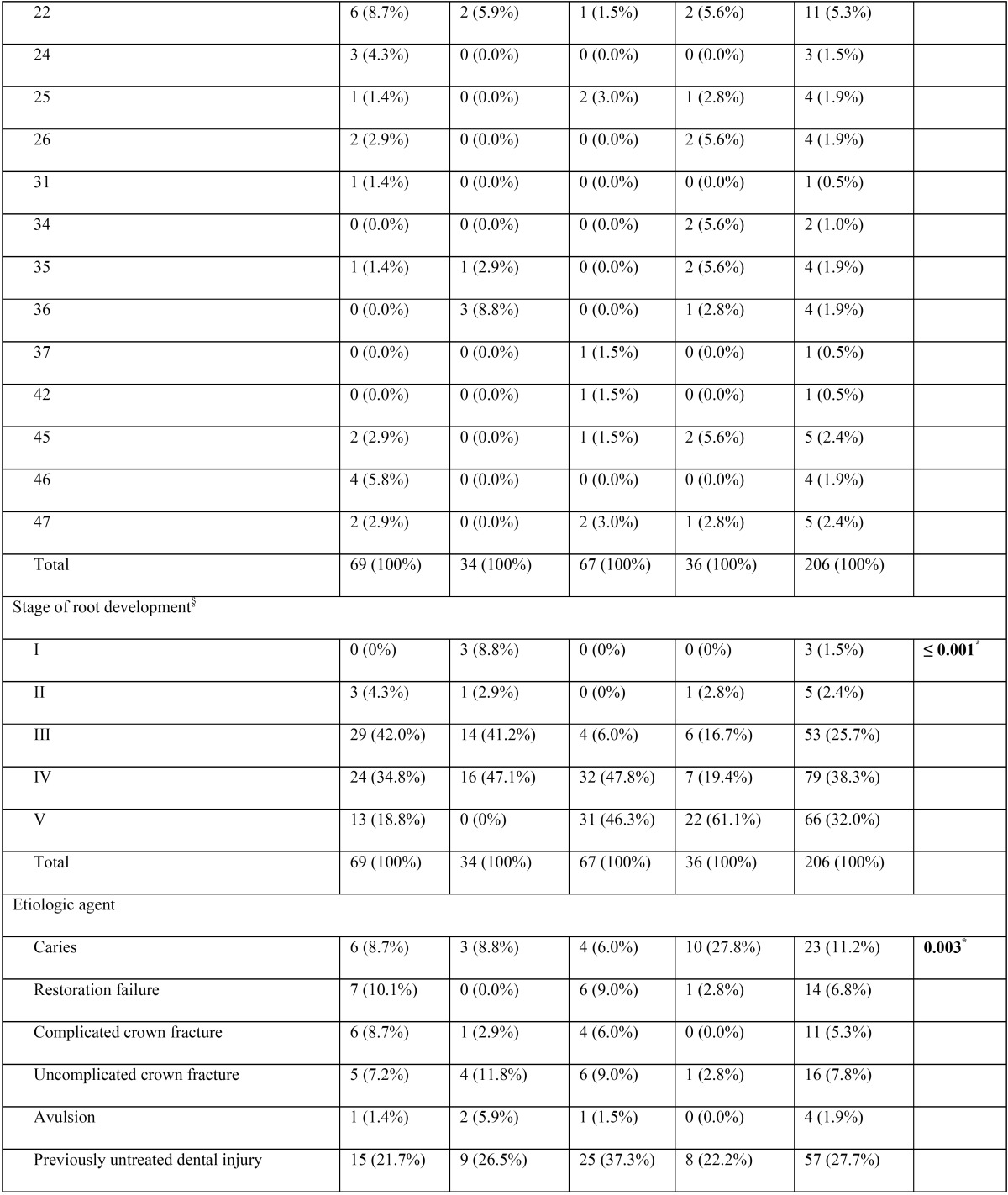
Distribution of the variables (n = 14) according to the endodontic technique modality applied.

**Table 1 continue-1:**
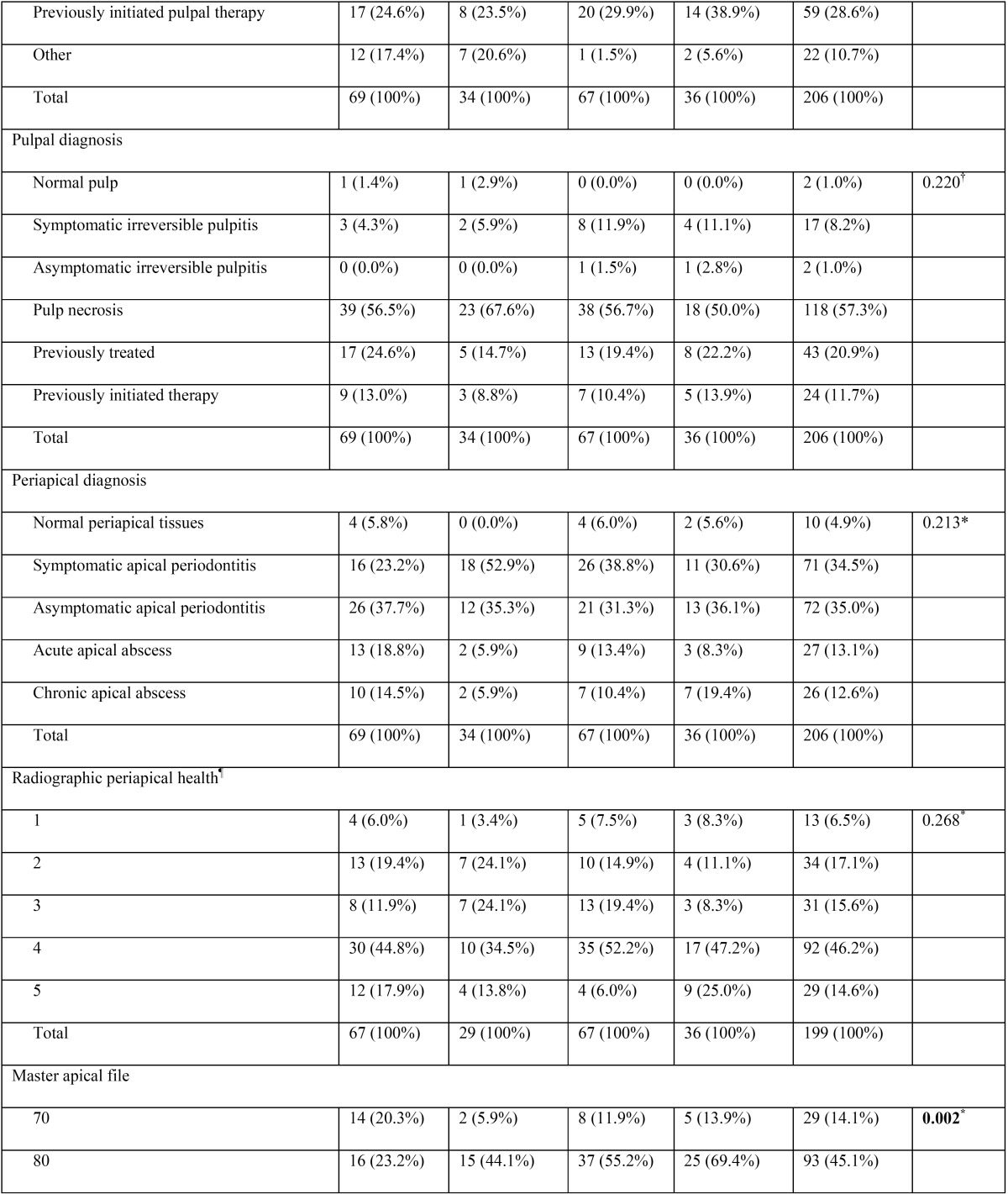
Distribution of the variables (n = 14) according to the endodontic technique modality applied.

**Table 1 continue-2:**
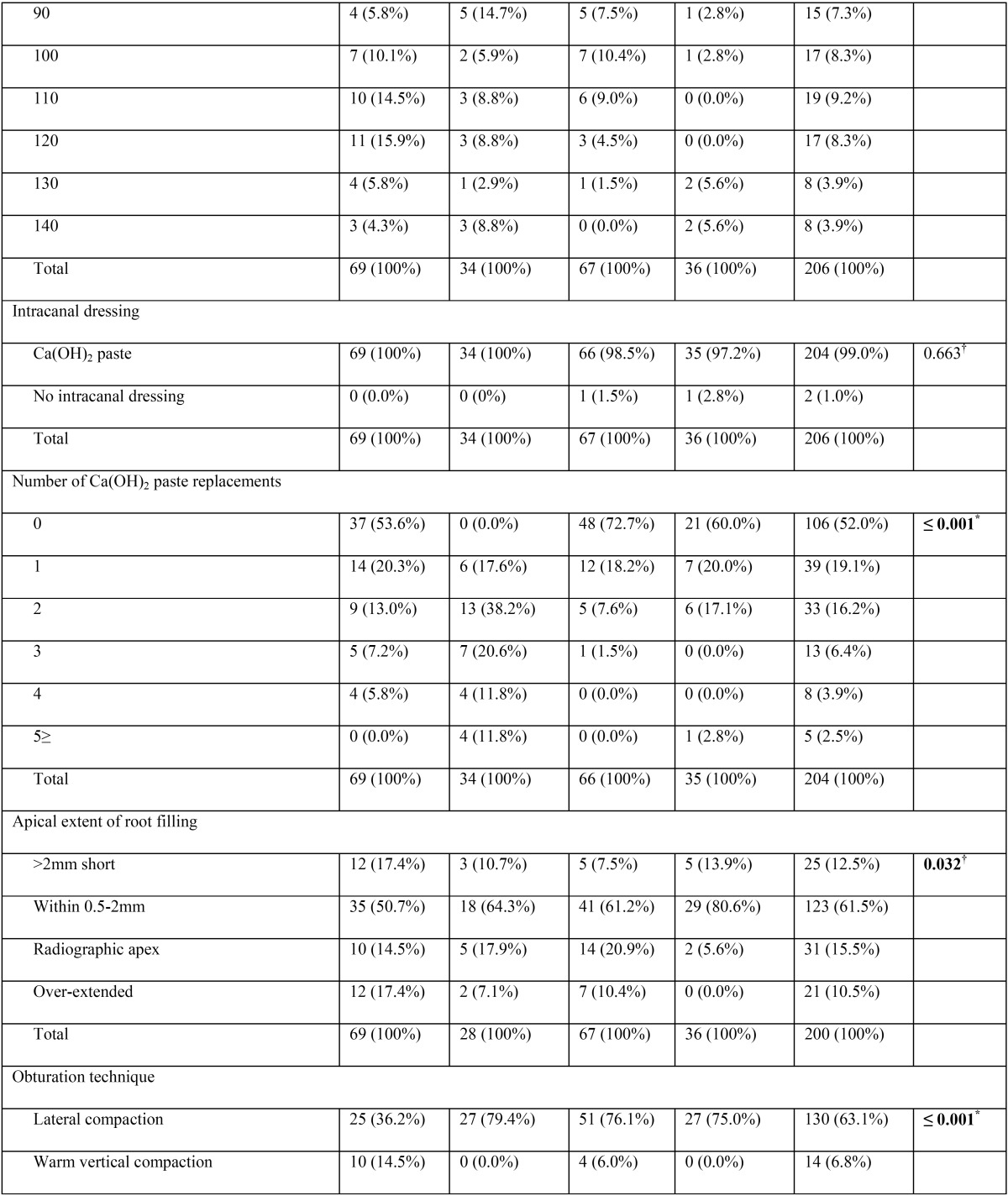
Distribution of the variables (n = 14) according to the endodontic technique modality applied.

**Table 1 continue-3:**
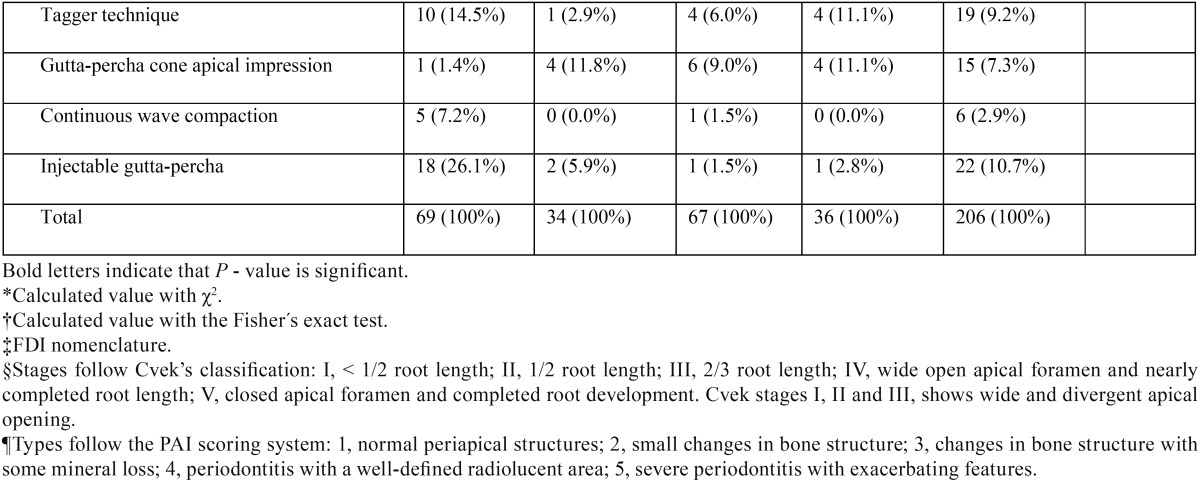
Distribution of the variables (n = 14) according to the endodontic technique modality applied.

The average age was 21.1 years (range 7 - 83 years), with the largest number of cases in the 10 to 14 year old age group (40.6%). Cases related to mature teeth or in patients ≥ 20 years of age were also well-represented (38.0%). Although the 206 cases were distributed among 20 different teeth, a predilection was observed for those localized to the maxilla (86.9%), particularly the maxillary central incisors (68.8%). The principal etiologic agent was dental trauma in 42.7% of cases (previously untreated dental injury = 27.7%, uncomplicated crown fracture = 7.8%, complicated crown fracture = 5.3%, and avulsion = 1.9%), followed by previously initiated pulpal therapy (28.6%) and caries (11.2%). During the calibration process for the variable radiographic periapical health analysis, the inter-observer concordance was k = 0.793, which indicate substantial concordance. The analysis of this variable was limited to 199 cases due to the exclusion of seven cases that presented tooth displacement resulting from trauma six months prior to clinical presentation (avulsion) or those involved in active orthodontic treatment. In accordance with the PAI classification, 23.6% of the cases were considered healthy periapical tissues (scores 1 and 2), and 76.4% were considered non-healthy periapical tissues (scores 3, 4 and 5). The most frequently observed radiographic periapical health score was 4 (periodontitis with a well-defined radiolucent area) in 46.2% of the cases examined.

All cases studied were distributed across four different endodontic therapies as follows: technique 1 (MTA) = 69, technique 2 (CHR) = 34, technique 3 (GTP) = 67, and technique 4 (CH-G) = 36. Eight different master apical file sizes were used, although in the second series, only two sizes were used in more than half of the cases (#70 = 14.1%, #80 = 45.1%). Ca(OH)2 paste was used in 99% of the cases as an intracanal dressing, with a predilection for 0 paste replacement (52%). However, for technique 2 (CHR), 7.71 months were needed on average to complete the procedure, which included a mean of two paste replacements (38.2%). For the apical extent of root filling, six cases were discarded (all of them associated with technique 2) due to a lack of post-operative radiographs in the dental record or the presence of poor-quality radiographs in which the obturation final limit could not be reliably determined. A total of 148 cases (74%) were filled within the root canal system, and 52 cases (26%) were filled outside. Furthermore, 123 cases (61.5%) had ideal apical levels (within 0.5 - 2mm). However, lateral compaction was the most frequently used obturation technique (63.1%).

When comparing the percentage distribution of the 14 variables versus the four different techniques ( Table 1), significant differences were observed for age (*P* = 0.0013), stage of root development (*P* ≤ 0.001), etiologic agent (*P* = 0.003), master apical file (*P* = 0.002), number of Ca(OH)2 paste replacements (*P* ≤ 0.001), apical extent of root filling (*P* = 0.032), and obturation technique (*P* ≤ 0.001).

-Stage of root development versus other variables

Teeth with necrotic pulps, open apices and intermediate root development (stage III) showed a predilection for techniques 1 (MTA) and 2 (CHR) (81.1% and 50.6%, respectively), which was statistically significant according to the χ2 analysis (*P* ≤ 0.001) ( Table 1). Regarding the correlation between the stages of root development versus the other 13 variables in this study, five variables showed statistically significant results ( Table 2). Therefore, it can be assumed that the distribution of these variables were not the same across the five degrees of radicular formation and were biased towards certain stages of root development.

**Table 2:**
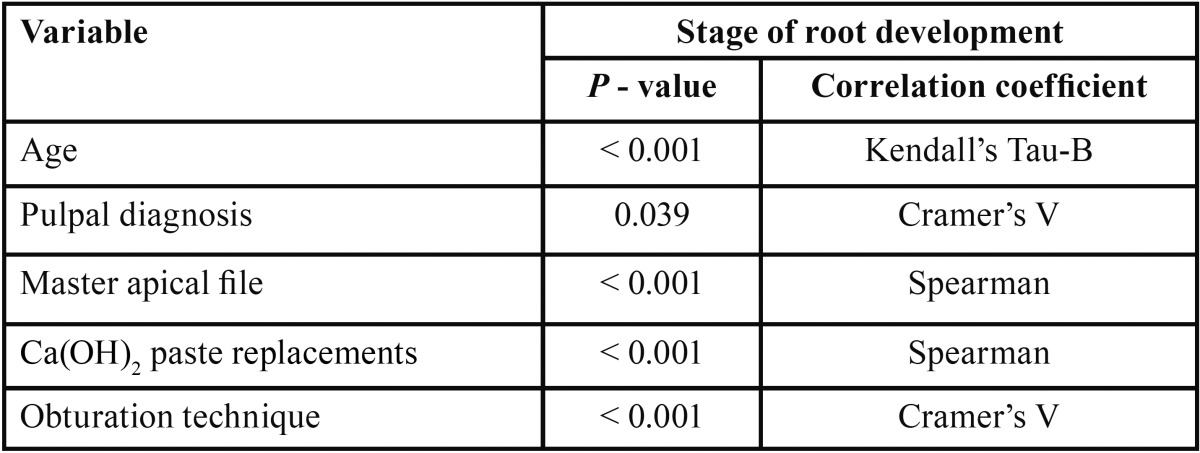
Summary of variables that showed significant correlation (*P*) with the stage of root development.

## Discussion

This epidemiological study analyzed the distribution of different root canal techniques performed in permanent teeth with necrotic pulps and open apices according to the stage of root development by endodontic residents in an institutional environment over a period of 14 years. This type of study presents inherent limitations, such as the fact that the results are not representative of Guadalajara’s population, and this analysis can therefore lead to biased data. Nonetheless, the obtained epidemiological information can be useful to clarify etiologic hypotheses or improve available techniques for management of these cases (18).

Typically, the clinical and radiographic diagnosis of teeth with open apices and necrotic pulp is based on a dichotomous option (yes / no). However, the term “open apex” could not be grouped as a single entity. Open apices include a wide variety of cellular changes and radicular morphologies throughout all phases of root development (19). When a tooth remains more immature, it becomes more difficult to mechanically clean the pulpal space, the risk of foreign body extrusion inside the periapical tissues increases due to the lack of apical constriction, and the presence of apical divergent anatomy (20,21). Although several classification systems for radicular formations have been proposed (13-15), the Cvek (11) classification criteria were used in this study due to the didactic radiographic characteristics of this system, which allows for better clinical application than that used in the other classification schemes. Nonetheless, the radiographic interpretation of permanent teeth with open apeices is complicated. Determination of the stage of root development should be performed by at least two experienced clinicians who have been previously calibrated (22), as in this study. Despite this consideration, 32% of the cases included were initially classified as teeth with mature apices (Cvek stage V), when in fact they were teeth with open apices or master apical files sizes ≥ #70.

The results of this study confirmed that the 10 to 14 year old age group was the group most commonly submitted to treatment (40.6%), as reported in previous studies (23,24). However, these procedures are not exclusive to young patients with immature roots. Completely formed teeth can suffer alteration in the terminal portion of the root by pathological (2,3) or iatrogenic (4) factors and develop open apices. The current study revealed 38.0% of procedures were performed in patients ≥ 20 years of age). However, teeth with fragile, irregular and divergent apical morphologies, such as Cvek stages I and II, were uncommon (1.5% and 2.4%, respectively) and may be considered to be unusual findings.

The distribution by sex did not show any significant tendency toward males or females (female = 53.6%, male = 46.4%). Theses results were inconsistent with traditional presumptions, which suggested that males are three times more predisposed than females to developing teeth with open apices and necrotic pulp (23,25), mainly due to masculine behaviors. These results are in alignment with a reported reduction in this male bias due to the increasing participation of women in contact sports (24,26). Additionally, the results from this study coincide with literature reports describing dento-alveolar trauma as the principal cause of open apices and necrotic pulp, with a noticeable predilection for teeth located in the maxilla, especially the maxillary central incisor (23,24,27,28).

For many years, teeth with necrotic pulps and open apices were submitted to complex endodontics treatments modalities regardless of the stage of root development, such as the apical adaptation of a gutta-percha cone through a softened filling technique, with (29,30) or without (31,32) a plug containing Ca(OH)2 powder. Actually, apexification procedures are often selected for these cases. Among the most commonly used apexification techniques is the development of a hard tissue apical barrier through periodic calcium hydroxide Ca(OH)2 paste replacements (33) or the creation of an MTA apical barrier (34,35). In terms of periapical tissue healing, all previous techniques show high success rates (36,37), although due to its technical advantages and higher bio-compatibility the MTA apical barrier is considered the first treatment option (38,39). However, one of the major challenges during the management of incompletely formed roots, is control of the apical limit of obturation due to the absence of natural apical constriction. Despite the above-mentioned studies, the results of this study showed that 74% of the cases were filled within the root canal system, with 123 cases (61.5%) filled to an ideal apical extent (within 0.5 - 2mm) regardless of the technique used. The aforementioned observations could be related to the stage of root development. The majority of cases required a master apical file, which makes the manipulation of the apical portion easier (master apical file #70 = 14.1%, #80 = 45.1%). It can be concluded that the four apexification techniques were reliable and secure in terms of the apical extent of root filling.

During analysis of the correlations between the stage of root development and the diverse treatments applied, the gutta-percha filling with or without a plug of Ca(OH)2 powder was typically used in partially or completely mature teeth (Cvek stages IV and V). This is related to the accidental creation of the open apex during the instrumentation phase. In contrast, in teeth with intermediate root development (Cvek stage III), which are routinely easy to detect during pre-operative diagnosis, this approach allows for the selection of an apexification technique, such as the MTA apical barrier or Ca(OH)2 replacements. All this results were statistically significant (*P* ≤ 0.001) ( Table 1).

In conclusions, the results of this study demonstrated that the finding of permanent teeth with necrotic pulp and open apices is not exclusive to young patients with an immature apex; such teeth can also be found in adult patients with open apices. Moreover, teeth with fragile, irregular and divergent apical morphologies, such as Cvek’s stages I and II, were uncommon and may be considered to be unusual findings. The four different procedures were considered reliable despite the stage of root development and the fact that the treatments were performed by endodontic residents. However, it is necessary to perform randomized controlled trials to confirm long-term clinical and radiographic success.
